# One Step Ahead: Herpesviruses Light the Way to Understanding Interferon-Stimulated Genes (ISGs)

**DOI:** 10.3389/fmicb.2020.00124

**Published:** 2020-02-07

**Authors:** A. Cristina Gonzalez-Perez, Markus Stempel, Baca Chan, Melanie M. Brinkmann

**Affiliations:** ^1^Viral Immune Modulation Research Group, Helmholtz Centre for Infection Research, Braunschweig, Germany; ^2^Institute of Genetics, Technische Universität Braunschweig, Braunschweig, Germany; ^3^Viral Genomics Group, Institute for Respiratory Health, The University of Western Australia, Perth, WA, Australia

**Keywords:** ISG, interferon, herpesvirus, immune evasion, innate immunity, HSV-1, HCMV, KSHV

## Abstract

The host immune system is engaged in a constant battle with microorganisms, with the immediate detection of pathogenic invasion and subsequent signalling acting as crucial deterrents against the establishment of a successful infection. For this purpose, cells are equipped with a variety of sensors called pattern recognition receptors (PRR), which rapidly detect intruders leading to the expression of antiviral type I interferons (IFN). Type I IFN are crucial cytokines which exert their biological effects through the induction of hundreds of IFN-stimulated genes (ISGs). The expression profile of these ISGs varies depending on the virus. For a small subset of ISGs, their anti- or even proviral effects have been revealed, however, the vast majority are uncharacterised. The spotlight is now on herpesviruses, with their large coding capacity and long co-evolution with their hosts, as a key to understanding the impact of ISGs during viral infection. Studies are emerging which have identified multiple herpesviral antagonists specifically targeting ISGs, hinting at the significant role these proteins must play in host defence against viral infection, with the promise of more to come. In this review, we will discuss the current knowledge of the complex interplay between ISGs and human herpesviruses: the antiviral role of selected ISGs during herpesviral infections, how herpesviruses antagonise these ISGs and, in some cases, even exploit them to benefit viral infection.

## Introduction

The *Herpesviridae* is a family of large, structurally complex viruses with double-stranded DNA genomes. This family is classified into three subfamilies according to biological and genomic similarities: *alphaherpesvirinae*, *betaherpesvirinae*, and *gammaherpesvirinae* (Pellett and Roizman, 2007). Several viruses with significant medical relevance are represented in this family, which cause a series of maladies ranging from cold sores or fever blisters to a variety of human cancers. A distinctive feature of herpesviruses is their ability to establish lifelong latent infections, with infected individuals serving as reservoirs from which period reactivation leads to continual and anew transmission to naive hosts.

Herpesviruses are known for the impressive toolbox they have evolved to circumvent the host’s immune response. Throughout the lifelong coexistence with their hosts, herpesviruses antagonise the immune response at every level: the signalling pathways downstream of pattern recognition receptors (PRR) (reviewed in Liu et al., 2019; Stempel et al., 2019) and the IFNα/β receptor (IFNAR) (Zimmermann et al., 2005), Natural Killer cell responses (reviewed in De Pelsmaeker et al., 2018), the complement system (reviewed in Stoermer and Morrison, 2011) and the adaptive immune response (reviewed in Smith and Khanna, 2013). However, our understanding of the interplay between herpesviruses and the interferon-stimulated gene (ISG) network is only in its infancy. So far, more than 380 human ISGs, with their functions ranging from sensors, cytokines or transcription factors, to proapoptotic proteins or negative regulators, have been tested for their ability to inhibit the replication of a panel of RNA viruses, revealing that different viruses are targeted by unique sets of ISGs (Schoggins et al., 2011). Such a screen has not been performed for the different members of the *Herpesviridae*, however, recent studies have identified multiple herpesviral antagonists which target ISGs, showcasing the importance of ISGs in combating herpesviral infection.

**FIGURE 1:**
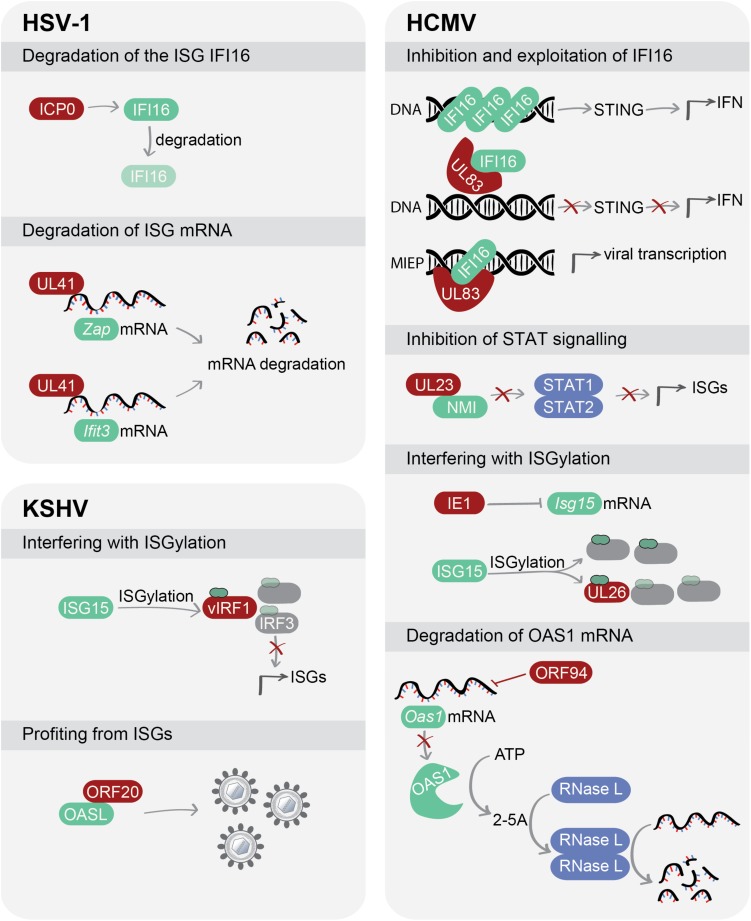
Herpesviruses use a variety of strategies to manipulate ISGs. Viral proteins can interfere with protein expression and stability of ISGs, inhibit signalling pathways exerted by ISGs or, in some cases, exploit ISGs for their own benefit. Viral proteins are depicted in red, while ISGs are shown in green. Abbreviations: HSV-1, herpes simplex virus type 1; HCMV, human cytomegalovirus; KSHV, Kaposi’s sarcoma-associated herpesvirus; IFI16, gamma-interferon-inducible protein 16; STING, stimulator of interferon genes; IFN, interferon; ZAP, zinc finger CCCH-type antiviral protein; IFIT3, interferon-induced protein with tetratricopeptide repeats 3; NMI, N-myc-interactor; STAT1/2, signal transducer and activator of transcription 1/2; ISG15, interferon-stimulated gene 15; IRF, interferon regulatory factor; OASL, 2′-5′-Oligoadenylate synthetase like; OAS1, 2′-5′-Oligoadenylate synthetase 1; RNase L, ribonuclease L.

In this review, we will discuss the current knowledge regarding the complex interaction between ISGs and human herpesviruses and highlight how each subfamily of human herpesviruses has evolved unique mechanisms to counteract ISGs or, in some cases, even exploit ISGs to the advantage of the virus (Figure 1).

## How It All Starts: ISGs Enter the Game

The DNA sensing pathway mediated by the PRR cyclic GMP-AMP synthase (cGAS) and gamma-interferon-inducible protein 16 (IFI16) is crucial for the initial immune response to herpesviral infection in many cell types (Ablasser et al., 2013; Li et al., 2013; Wu et al., 2015; Paijo et al., 2016). The DNA sensors cGAS and IFI16 bind to viral and aberrantly localised cellular DNA. This interaction activates a signalling cascade through the adaptor protein stimulator of interferon genes (STING) and TANK-binding kinase 1 (TBK1), thereby activating the transcription factors interferon regulatory factor 3 (IRF3) and nuclear factor kappa-light-chain-enhancer of activated B cells (NF-κB) (reviewed in Chen et al., 2016). This leads to the induction of type I interferons (IFN), which exert their activity in an autocrine and paracrine manner. By binding to the interferon-α/β receptor (IFNAR), these cytokines lead to phosphorylation and activation of the transcription factors signal transducers and activators of transcription, STAT1 and STAT2, recruiting IRF9 into the complex which then translocates to the nucleus, resulting in ISG expression (reviewed in Schneider et al., 2014). Another class of ISGs, known as non-canonical ISGs, are activated directly by IRF3 in the absence of type I IFN (Schoggins et al., 2014). For a third class of proteins classified as ISGs, the presence of IRF3 or type I IFN is not an absolute prerequisite for their expression, since they are already expressed basally or their expression is induced by other pathways, i.e., NF-κB signalling (reviewed in Schoggins, 2019). Thus, due to this complexity and the wide range of functions that ISGs can exert, studying how herpesviruses manipulate ISGs to their advantage serves as a window into a greater understanding of the myriad of ISGs and their role in innate immunity. Similar to the studies on ISGs and RNA viruses (Schoggins et al., 2011), studies identifying how herpesviruses inhibit or exploit the function of ISGs may reveal the essential nature of the role these ISGs play in viral defence.

## When Herpesviruses Win: Escaping the Antiviral Effects of ISGs

### Herpes Simplex Virus 1

Herpes simplex virus 1 (HSV-1) belongs to the *Alphaherpesvirinae* subfamily. HSV-1 establishes a primary infection in mucosal epithelia and a latent infection in the ganglia of sensory nerves. This infection, as in the case of all herpesviruses, can be asymptomatic, but it may also present as acute gingivostomatitis. Furthermore, HSV-1 can lead to serious illnesses like ophthalmic infections, meningitis or encephalitis (Pellett and Roizman, 2007). Recently, HSV-1 has also been associated as a major risk factor for Alzheimer’s disease (Itzhaki, 2018).

#### Targeting DNA Sensing: ICP0 Degrades the ISG IFI16

Herpesviruses replicate in the nucleus of their host cells. The ISG IFI16 is a cellular DNA sensor localized in the nucleus of many cell types (Unterholzner et al., 2010; Duan et al., 2011; Veeranki and Choubey, 2012; Jonsson et al., 2017). Orzalli et al. (2012) demonstrated that HSV-1 targets the IFI16 protein. During infection, when HSV-1 expresses the immediate-early viral protein ICP0 in the nucleus of human foreskin fibroblasts (HFF), IFI16 undergoes a change in its location and is continuously degraded. However, another study found that the expression of ICP0 alone is neither sufficient nor necessary for degradation of IFI16 in the tumor-derived cell line U-2 OS, since infection with an ICP0-null mutant still resulted in IFI16 degradation (Cuchet-Lourenco et al., 2013). A follow-up study by Orzalli et al. (2016) clarified this discrepancy by showing that IFI16 protein levels decrease upon HSV-1 infection in HFF, keratinocytes (NOK), and HeLa cells, but not in the U-2 OS cell line, and additionally discovered that ICP0 is not the only protein involved in IFI16 degradation (Table 1). This suggests that the role of ICP0 for IFI16 degradation is dependent on the cell type and other cellular or viral factors contributing to IFI16 stability (Kalamvoki and Roizman, 2014).

**TABLE 1:** Viral antagonists of interferon-stimulated genes (ISGs).

**Virus**	**Strain**	**Viral antagonist**	**Target ISG**	**Cell type**	**Viral evasion strategy**	**References**
**HSV-1**	KOS	ICP0	IFI16	HFF	Degrades IFI16	Orzalli et al., 2012
	17+	ICP0	IFI16	U-2 OS	Does not degrade IFI16	Cuchet-Lourenco et al., 2013
	KOS, 17, F	UL41 (vhs)	IFI16	HFF NOK, HeLa, U-2 OS	ICP0 degrades IFI16 in a cell-type specific manner; UL41 also reduces protein levels of IFI16 (presumably by RNA degradation)	Orzalli et al., 2016
	F	UL41	ZAP	293Trex-hZAPL/S	Degrades ZAP mRNA through its endoribonuclease activity, preferentially binds ARE motifs	Su et al., 2015
	F	UL41	IFIT3	293T	Decreases IFIT3 expression levels by degrading IFIT3 mRNA, does not target IFIT1 or IFIT2	Jiang et al., 2016
**HCMV**	AD169	UL83 (pp65)	IFI16	HFF	Interacts with IFI16 to block its oligomerisation and prevents signalling; promotes transcription of immediate early genes by exploiting the binding capacity of IFI16 to DNA	Li et al., 2013
	TB40/E					Biolatti et al., 2016
	AD169					Cristea et al., 2010
	Towne (BAC-derived)	UL23	NMI	U251	Inhibits ISG transcription by binding to NMI and disrupting its association with STAT1	Feng et al., 2018
	Towne	IE1 (UL123)	ISG15	HF	Inhibits HCMV-induced ISG15 expression and thereby prevents ISGylation	Kim et al., 2016
	AD169	UL26	ISG15	HF	Reduces the accumulation of ISGylated proteins by acting as a decoy target for ISG15	Kim et al., 2016
	Towne	ORF94 (UL126a)	OAS1	HF	Inhibits mRNA and protein expression of OAS1, leading to reduced viral RNA degradation	Tan et al., 2011
**KSHV**	iSLK.219 harbouring rKSHV.219*	vIRF1	ISG15	293, 293-TLR, BCBL PEL, iSLK.219	Reduces ISGylation of cellular target proteins, leading to IRF3 instability and decreased ISG transcription; acts as a decoy target for ISG15	Jacobs et al., 2015
	HuARLT2 harbouring rKSHV.219*	ORF20	OASL	293T, HeLa, HFF, HuARLT2-rKSHV.219	ORF20 and OASL interact; ORF20 increases RIG-I dependent OASL expression; OASL and ORF20 concomitantly enhance KSHV infection	Bussey et al., 2018

*These studies used iSLK or HuARLT2 cells that were latently infected with recombinant rKSHV.219 (Vieira and O’Hearn, 2004; Myoung and Ganem, 2011; Lipps et al., 2017). HFF, human foreskin fibroblasts; HF, human fibroblasts.

#### Degrading ISG mRNA: UL41 Counteracts ZAP and IFIT3

The HSV-1 tegument protein UL41, also known as virion host shutoff (vhs) protein, is an endoribonuclease that degrades mRNA (Everly et al., 2002; Page and Read, 2010). It is proposed that viral and cellular mRNAs containing AU-rich elements (ARE) in the 3′-untranslated region (3′-UTR) are the preferred target for UL41 (Esclatine et al., 2004; Taddeo and Roizman, 2006). Since ARE regions are frequently present in transcripts connected to the immune response, including interferons and chemokines (Bakheet et al., 2001), UL41 could potentially target a broad spectrum of transcripts. To date, the zinc finger CCCH-type antiviral protein 1 (ZAP) and the ISG interferon-induced protein with tetratricopeptide repeats 3 (IFIT3) are two ISGs that contain ARE in their 3′-UTR which have been shown to be incapacitated by UL41 (Figure 1).

The zinc finger CCCH-type antiviral protein is a non-canonical ISG (Schoggins et al., 2014), meaning that its expression can also be induced in the absence of type I IFN production. ZAP exerts antiviral activity against a diverse range of viruses such as retroviruses, alphaviruses, filoviruses, hepatitis B virus and Japanese encephalitis virus by binding to RNA and indirectly mediating its degradation (Bick et al., 2003; Muller et al., 2007; Zhu et al., 2011; Mao et al., 2013; Takata et al., 2017; Chiu et al., 2018). However, ZAP fails to control other viruses, e.g., influenza A virus (Liu et al., 2015; Tang et al., 2017) or enterovirus A71 (Xie et al., 2018).

In the case of HSV-1, ectopic expression of both rat and human forms of ZAP does not affect HSV-1 infection (Bick et al., 2003), which suggested that a viral antagonist may counteract the antiviral activity of ZAP. Accordingly, a luciferase-based assay in 293T cells identified the HSV-1 UL41 protein as a ZAP antagonist (Su et al., 2015). In accordance with previous observations regarding the nuclease activity of UL41, this viral protein was shown to degrade ZAP mRNA during HSV-1 infection. Correspondingly, growth of a mutant virus lacking UL41 expression was impaired in the presence of ZAP (Su et al., 2015).

Similarly, IFIT3 was reported to have no effect on HSV-1 infection (Jiang et al., 2016). As for ZAP, human IFIT proteins with the family members IFIT1, IFIT2, and IFIT3 belong to the subgroup of non-canonical ISGs (Schoggins et al., 2014). IFIT3 mediates the association of TBK1 with mitochondrial antiviral-signalling protein (MAVS) at the mitochondria (Liu et al., 2011), enhancing the MAVS-TBK1 signalling axis. Notably, IFIT3 inhibits the replication of HSV-1 lacking UL41 expression, underlining the importance of UL41 in evading the antiviral effect of IFIT3. The authors showed that UL41 degrades IFIT3 mRNA, but not that of IFIT1 or IFIT2 (Jiang et al., 2016), indicating that HSV-1 may specifically target IFIT3 to prevent the MAVS-TBK1 association, thus suppressing downstream signalling.

### Human Cytomegalovirus

Human cytomegalovirus (HCMV), also called human herpesvirus 5 (HHV-5), is a member of the *Betaherpesvirinae* subfamily. HCMV usually causes only mild disease in immunocompetent individuals. However, in immunosuppressed individuals such as AIDS or transplant patients, HCMV infection can cause severe complications (reviewed in Arvin and National Center for Biotechnology, 2007). HCMV infection during pregnancy can cause long-term sequelae in newborns, such as hearing loss, vision abnormalities, microcephaly or global development delays.

#### Targeting DNA Sensing: UL83 Hijacks the ISG IFI16

Human cytomegalovirus, as for HSV-1, interferes with DNA sensing by targeting IFI16 via the UL83 encoded tegument protein pp65. Upon HCMV infection, IFI16 is activated in the nucleus and undergoes oligomerisation, which is a prerequisite for it to promote the immune response (Cristea et al., 2010; Li et al., 2013). Accordingly, siRNA-mediated silencing of IFI16 dampens cytokine transcription in response to HCMV infection (Li et al., 2013). However, UL83 prevents IFI16 oligomerisation, thus disarming the antiviral effect of IFI16 during HCMV infection (Li et al., 2013). UL83 even goes a step further in its manipulation of host responses: it exploits the binding capacity of IFI16 to DNA in order to form a complex with the major immediate early promoter (MIEP) of HCMV, thereby triggering viral transcription in the early stages of infection (Cristea et al., 2010; Biolatti et al., 2016). In this manner, UL83 not only prevents the antiviral activity of IFI16 but also hijacks it to promote HCMV gene expression (Figure 1). This viral protein serves as a stellar example of the resourcefulness of herpesviruses in encoding a protein that can simultaneously inhibit a host antiviral strategy while exploiting this same host response factor to promote its own replication.

#### Fooling the Type I IFN Response: UL23 Inhibits ISG Transcription by Targeting the ISG NMI

The ISG N-myc interactor (NMI) interacts with all STATs, except STAT2, and enhances the recruitment of co-activators, such as the transcription factors CREB-binding protein (CBP)/p300, to the STAT complex. NMI specifically modulates IFN-induced signalling to foster efficient STAT-dependent transcription (Zhu et al., 1999). Recently, the HCMV tegument protein UL23 was reported to inhibit the transcription of ISGs by targeting NMI. Through a yeast two-hybrid screen, NMI was identified as an interacting partner of UL23, which was confirmed by co-immunoprecipitation in HCMV-infected U251 cells (Feng et al., 2018). Using a combination of immunofluorescence, cell fractionation and immunoblotting, the authors showed that the binding of UL23 to NMI disrupts its association with STAT1, thereby preventing the translocation of both proteins to the nucleus (Figure 1). Infection of U251 cells with an HCMV UL23-null mutant resulted in enhanced transcription of antiviral genes and controlled viral replication.

#### HCMV Finds Ways to Avoid ISGylation: Both IE1 and UL26 Target ISG15

Interferon-stimulated gene 15 (ISG15) encodes an ubiquitin-like protein that, in a similar way to ubiquitin, covalently conjugates to lysine residues, thereby regulating protein function (reviewed in Jeon et al., 2010). ISG15 modification is known as ISGylation, which marks proteins for either degradation or stabilisation. ISG15 is one of the most abundantly produced transcripts upon induction of the type I IFN response (Der et al., 1998; Potter et al., 1999) and exerts antiviral effects against DNA and RNA viruses (Lenschow, 2010; Morales and Lenschow, 2013).

Human cytomegalovirus infection induces ISG15 expression, which, through ISGylation, inhibits viral replication (Kim et al., 2016). HCMV employs two proteins with two separate strategies to evade this process (Figure 1). First, the viral immediate-early protein IE1 suppresses ISG15 transcription (Kim et al., 2016). However, this effect is only partial, and therefore some ISG15 protein is still expressed to carry out ISGylation, which is protected from the antagonistic activity of IE1. To counteract this remnant ISG15, HCMV expresses the tegument protein UL26, which reduces the accumulation of other viral ISGylated proteins by acting as a decoy for ISGylation itself (Kim et al., 2016). UL26 is known as an antagonist of the NF-κB pathway (Mathers et al., 2014), but ISGylated UL26 can no longer antagonise NF-κB signalling (Kim et al., 2016), suggesting that the virus sacrifices one of its own proteins to avoid ISGylation of other viral proteins. Why UL26 is more prone to ISGylation compared to other viral proteins, and the impact of the loss of its effect on NF-κB signalling during HCMV infection remains unclear at this stage. This in turn raises the question of whether the dominant role of UL26 is to inhibit NF-κB signalling or to act as an ISGylation decoy, since these seem to be opposing functions.

#### Targeting an Essential Player of the Innate Immune Response: ORF94 Against the ISG OAS1

Human cytomegalovirus expresses several genes during latency to avoid immune recognition of infected cells (Jenkins et al., 2004; Cheung et al., 2009), the so-called CMV latency-associated transcripts (CLTs). These products are also expressed during lytic HCMV infection. HCMV ORF94 (also known as UL126a) is one such transcript, and its localization in the nucleus suggests a potential role in cellular gene regulation (White et al., 2000). ORF94 was shown to inhibit both the transcription and translation of the ISG 2′-5′-oligoadenylate synthetase 1 (OAS1) (Tan et al., 2011). OAS1, together with OAS2, OAS3, OAS-like (OASL), and cGAS, forms the OAS family (Justesen et al., 2000). Upon detection of double stranded RNA (dsRNA), OAS1-3 proteins are activated and oligomerise ATP into 2′,5′-linked oligoadenylate products (2-5A). This leads to the activation of endoribonuclease L (RNase L), which in turn degrades viral and cellular RNA. Thus, expression of HCMV ORF94 reduces OAS mRNA and protein levels and consequently the formation of 2-5A during productive infection in human fibroblasts (Figure 1; Tan et al., 2011). However, as ORF94 is expressed in both the productive and latent phases of HCMV infection, it could potentially contribute to latency by modulating the immune response, which would be an intriguing avenue of further research.

### Kaposi’s Sarcoma-Associated Herpesvirus

Kaposi’s sarcoma-associated herpesvirus (KSHV), also called human herpesvirus 8 (HHV-8), belongs to the *Gammaherpesvirinae* subfamily. KSHV is one of the seven known human oncoviruses. It can cause multiple malignancies, namely Kaposi’s sarcoma, primary effusion lymphoma, multicentric Castleman’s disease, or KSHV inflammatory cytokine syndrome (Chang et al., 1994; Ablashi et al., 2002; Ganem, 2006).

#### Targeting ISGylation: vIRF1 and ISG15

Kaposi’s sarcoma-associated herpesvirus encodes four viral homologs of cellular interferon regulatory factors (vIRFs) (Jacobs and Damania, 2011). In 2013, Jacobs et al. (2013) showed that vIRF1 inhibits the type I IFN response. By performing affinity purification coupled to mass spectrometry with cells expressing vIRF1 and in which TLR3 signalling was activated, the authors identified the cellular ISG15 E3 ligase, HERC5, as an interaction partner of vIRF1 (Jacobs et al., 2015). HERC5 interacts with the C-terminus of vIRF1. Moreover, vIRF1 reduces total ISG15 conjugation levels on cellular target proteins, which in turn inhibits IRF3 function as it relies on ISGylation for stabilisation (Figure 1; Shi et al., 2010). Additionally, siRNA-mediated knockdown of ISG15 or HERC5 increases KSHV replication upon reactivation. Therefore, it is possible that vIRF1 negatively regulates ISGylation by interacting with HERC5, leading to a decrease in IRF3 stability and reduced transcription of ISGs. Interestingly, the authors observed by immunoprecipitation that vIRF1 is conjugated to ISG15 at multiple sites, suggesting a role as a viral ISGylation target similar to the HCMV protein UL26 (Kim et al., 2016), which may reflect a conservation of this function between herpesvirus subfamilies.

While KSHV vIRFs inhibit IFN signalling, type I IFN is not always detrimental for herpesviruses as it plays an important role for the maintenance of latency (Zhang et al., 2004; De Regge et al., 2010; Dag et al., 2014; Holzki et al., 2015). In line with these findings, vIRF2 has been recently described to manipulate the innate immune response. vIRF2 regulates the expression of 51 genes known to be involved in innate or intrinsic defences, boosting the formation of the antiviral cellular state to restrict KSHV early lytic protein expression and promote latency (Koch et al., 2019). This is an intriguing illustration of the fine-tuned balance between herpesviruses and their host, which dictates the outcome of the infection course.

#### Profiting From ISGs: ORF20 Fancies the ISG OASL

The OAS family member OASL shares a highly conserved N-terminal OAS-like domain with the OAS enzymes, but it lacks enzymatic activity and has a unique C-terminus composed of two ubiquitin-like domains (Hartmann et al., 1998). In addition, OASL binds dsRNA (Ibsen et al., 2015). OASL was identified as an ISG with targeted, but not broad antiviral specificity against a variety of RNA viruses (Schoggins et al., 2011, 2014). Its role for HSV-1 is more controversial - while one study observed no role for OASL on HSV-1 replication (Marques et al., 2008), another reported that OASL inhibited HSV-1 (Zhu et al., 2014).

We showed that the KSHV protein ORF20 interacts with OASL, presumably in the nucleoli given their subcellular localization (Bussey et al., 2018). Interestingly, stable expression of OASL enhances KSHV replication in an ORF20-dependent manner (Figure 1). Since both proteins interact with ribosomal proteins and co-sediment with ribosomal subunits, which are involved in the formation of active ribosomal complexes, ORF20 may manipulate OASL so that KSHV can seize control of the host translational machinery. However, further studies are needed to understand the mechanism by which KSHV ORF20 usurps OASL. It is worth noting that the expression of ORF20 in 293T cells specifically enhances OASL mRNA and protein levels. This may be congruent with the observation of a recent study that OASL negatively affects the DNA-binding ability of the DNA sensor cGAS (Ghosh et al., 2019), which is a crucial sensor of KSHV infection (Wu et al., 2015). Thus, enhanced levels of OASL during lytic KSHV replication may inhibit cGAS-mediated activation of the innate immune response and therefore provide a more conducive environment for infection.

## Final Remarks

The complex interaction between herpesviruses and their host is essential for the outcome of infection. In the case of ISGs, understanding the mechanisms by which herpesviruses manipulate these effectors gives an insight into both how viruses establish lifelong infections and the role that ISGs play in immune defence. The importance of ISGs for antiviral defence is indisputable, given that IFNAR knockout mice readily succumb to infection with herpesviruses (Strobl et al., 2005; Lenschow et al., 2007; Rasmussen et al., 2007). Interestingly, several studies reported only minor defects in mice lacking individual ISGs (Lenschow et al., 2007), supporting the notion that at least some ISGs may act in concert to exert their full effect, or the existence of ISGs with redundant functions. Moreover, a recent study revealed the complex network that ISGs create during viral infection, not just by binding to other ISGs, but also to many other cellular proteins (Hubel et al., 2019), adding an even greater level of complexity to the host immune response against infection.

We have only just crossed the starting line to understanding whether certain ISGs are proviral or antiviral in the context of herpesviral infections. This investigation into the role and mechanism of action of ISGs is challenging. Overexpression studies may give some valuable insights into the function of these ISGs. However, since viral infections induce the expression of multiple ISGs that may cooperate, studies on a single ISG may not reflect reality or at least may not reveal the full potential of the individual ISG tested. Ideally, tagged, endogenously expressed ISGs would be used for co-immunoprecipitation studies in infected cells to identify viral and/or cellular binding partners of them. To expand on these studies, analysis of single and combined ISG knockouts will help to determine whether ISGs have a proviral role, an antiviral role, or neither. Another point to consider is that some ISGs may have diverse functions in different cell types while other ISGs may be species-specific.

Herpesviruses are a very valuable tool in the endeavour to uncover the role that ISGs play in antiviral defence as they are highly adapted and have likely developed multiple antagonists (Table 1). However, viral antagonists can be friend or foe: while the function of ISGs may only be revealed in the absence of viral antagonists, these opponents may be key to our greater understanding of how cellular defence is regulated. Through our bid to decipher the intricacies of this complex interplay between herpesviruses and the tailored ISG response to individual infections, we may uncover novel targeted therapies against these masters of immune escape and manipulation.

## Author Contributions

AG-P conducted the literature research, critically analyzed the published data, planned the concept of the review with MB, prepared the table and figure, and wrote the manuscript. BC and MB wrote parts of the Introduction and Conclusion. MS, BC, and MB critically discussed and revised the manuscript together with AG-P.

## Conflict of Interest

The authors declare that the research was conducted in the absence of any commercial or financial relationships that could be construed as a potential conflict of interest.

## References

[B1] Ablashi D. V. Chatlynne L. G. Whitman J. E. Jr. Cesarman E. (2002). Spectrum of Kaposi’s sarcoma-associated herpesvirus, or human herpesvirus 8, diseases. *Clin. Microbiol. Rev.* 15 439–464. 12097251 10.1128/CMR.15.3.439-464.2002 PMC118087

[B2] Ablasser A. Goldeck M. Cavlar T. Deimling T. Witte G. Rohl I. (2013). cGAS produces a 2′-5′-linked cyclic dinucleotide second messenger that activates STING. *Nature* 498 380–384. 10.1038/nature12306 23722158 PMC4143541

[B3] Arvin A. M. National Center for Biotechnology (2007). *Human Herpesviruses Biology, therapy, and Immunoprophylaxis.* Cambridge: Cambridge University Press. 21348071

[B4] Bakheet T. Frevel M. Williams B. R. Greer W. Khabar K. S. (2001). ARED: human AU-rich element-containing mRNA database reveals an unexpectedly diverse functional repertoire of encoded proteins. *Nucleic Acids Res.* 29 246–254. 10.1093/nar/29.1.246 11125104 PMC29778

[B5] Bick M. J. Carroll J. W. Gao G. Goff S. P. Rice C. M. Macdonald M. R. (2003). Expression of the zinc-finger antiviral protein inhibits alphavirus replication. *J. Virol.* 77 11555–11562. 10.1128/jvi.77.21.11555-11562.2003 14557641 PMC229374

[B6] Biolatti M. Dell’oste V. Pautasso S. Von Einem J. Marschall M. Plachter B. (2016). Regulatory interaction between the cellular restriction factor IFI16 and Viral pp65 (pUL83) modulates viral gene expression and IFI16 Protein Stability. *J. Virol.* 90 8238–8250. 10.1128/JVI.00923-16 27384655 PMC5008087

[B7] Bussey K. A. Lau U. Schumann S. Gallo A. Osbelt L. Stempel M. (2018). The interferon-stimulated gene product oligoadenylate synthetase-like protein enhances replication of Kaposi’s sarcoma-associated herpesvirus (KSHV) and interacts with the KSHV ORF20 protein. *PLoS Pathog* 14:e1006937. 10.1371/journal.ppat.1006937 29499066 PMC5851652

[B8] Chang Y. Cesarman E. Pessin M. S. Lee F. Culpepper J. Knowles D. M. (1994). Identification of herpesvirus-like DNA sequences in AIDS-associated Kaposi’s sarcoma. *Science* 266 1865–1869. 10.1126/science.7997879 7997879

[B9] Chen Q. Sun L. Chen Z. J. (2016). Regulation and function of the cGAS-STING pathway of cytosolic DNA sensing. *Nat. Immunol.* 17 1142–1149. 10.1038/ni.3558 27648547

[B10] Cheung A. K. Gottlieb D. J. Plachter B. Pepperl-Klindworth S. Avdic S. Cunningham A. L. (2009). The role of the human cytomegalovirus UL111A gene in down-regulating CD4+ T-cell recognition of latently infected cells: implications for virus elimination during latency. *Blood* 114 4128–4137. 10.1182/blood-2008-12-197111 19706889

[B11] Chiu H. P. Chiu H. Yang C. F. Lee Y. L. Chiu F. L. Kuo H. C. (2018). Inhibition of Japanese encephalitis virus infection by the host zinc-finger antiviral protein. *PLoS Pathog* 14:e1007166. 10.1371/journal.ppat.1007166 30016363 PMC6049953

[B12] Cristea I. M. Moorman N. J. Terhune S. S. Cuevas C. D. O’keefe E. S. Rout M. P. (2010). Human cytomegalovirus pUL83 stimulates activity of the viral immediate-early promoter through its interaction with the cellular IFI16 protein. *J. Virol.* 84 7803–7814. 10.1128/JVI.00139-10 20504932 PMC2897612

[B13] Cuchet-Lourenco D. Anderson G. Sloan E. Orr A. Everett R. D. (2013). The viral ubiquitin ligase ICP0 is neither sufficient nor necessary for degradation of the cellular DNA sensor IFI16 during herpes simplex virus 1 infection. *J. Virol.* 87 13422–13432. 10.1128/JVI.02474-13 24089555 PMC3838218

[B14] Dag F. Dolken L. Holzki J. Drabig A. Weingartner A. Schwerk J. (2014). Reversible silencing of cytomegalovirus genomes by type I interferon governs virus latency. *PLoS Pathog* 10:e1003962. 10.1371/journal.ppat.1003962 24586165 PMC3930589

[B15] De Pelsmaeker S. Romero N. Vitale M. Favoreel H. W. (2018). Herpesvirus evasion of natural killer cells. *J. Virol.* 92 e1909–e1916. 10.1128/JVI.02105-17 PMC5952149 29540598

[B16] De Regge N. Van Opdenbosch N. Nauwynck H. J. Efstathiou S. Favoreel H. W. (2010). Interferon alpha induces establishment of alphaherpesvirus latency in sensory neurons in vitro. *PLoS One* 5:e13076. 10.1371/journal.pone.0013076 20927329 PMC2947521

[B17] Der S. D. Zhou A. Williams B. R. Silverman R. H. (1998). Identification of genes differentially regulated by interferon alpha, beta, or gamma using oligonucleotide arrays. *Proc. Natl. Acad. Sci. U.S.A.* 95 15623–15628. 10.1073/pnas.95.26.15623 9861020 PMC28094

[B18] Duan X. Ponomareva L. Veeranki S. Panchanathan R. Dickerson E. Choubey D. (2011). Differential roles for the interferon-inducible IFI16 and AIM2 innate immune sensors for cytosolic DNA in cellular senescence of human fibroblasts. *Mol. Cancer Res.* 9 589–602. 10.1158/1541-7786.MCR-10-0565 21471287 PMC3096691

[B19] Esclatine A. Taddeo B. Roizman B. (2004). The UL41 protein of herpes simplex virus mediates selective stabilization or degradation of cellular mRNAs. *Proc. Natl. Acad. Sci. U.S.A.* 101 18165–18170. 10.1073/pnas.0408272102 15596716 PMC539803

[B20] Everly D. N. Jr. Feng P. Mian I. S. Read G. S. (2002). mRNA degradation by the virion host shutoff (Vhs) protein of herpes simplex virus: genetic and biochemical evidence that Vhs is a nuclease. *J. Virol.* 76 8560–8571. 10.1128/jvi.76.17.8560-8571.2002 12163576 PMC136990

[B21] Feng L. Sheng J. Vu G. P. Liu Y. Foo C. Wu S. (2018). Human cytomegalovirus UL23 inhibits transcription of interferon-gamma stimulated genes and blocks antiviral interferon-gamma responses by interacting with human N-myc interactor protein. *PLoS Pathog* 14:e1006867. 10.1371/journal.ppat.1006867 29377960 PMC5805366

[B22] Ganem D. (2006). KSHV infection and the pathogenesis of Kaposi’s sarcoma. *Annu. Rev. Pathol.* 1 273–296. 18039116 10.1146/annurev.pathol.1.110304.100133

[B23] Ghosh A. Shao L. Sampath P. Zhao B. Patel N. V. Zhu J. (2019). Oligoadenylate-synthetase-family protein OASL inhibits activity of the DNA Sensor cGAS during DNA Virus Infection to Limit Interferon Production. *Immunity* 50 51.e5–63.e5. 10.1016/j.immuni.2018.12.013 30635239 PMC6342484

[B24] Hartmann R. Olsen H. S. Widder S. Jorgensen R. Justesen J. (1998). p59OASL, a 2′-5′ oligoadenylate synthetase like protein: a novel human gene related to the 2′-5′ oligoadenylate synthetase family. *Nucleic Acids Res.* 26 4121–4128. 10.1093/nar/26.18.4121 9722630 PMC147837

[B25] Holzki J. K. Dag F. Dekhtiarenko I. Rand U. Casalegno-Garduno R. Trittel S. (2015). Type I interferon released by myeloid dendritic cells reversibly impairs cytomegalovirus replication by inhibiting immediate early gene expression. *J. Virol.* 89 9886–9895. 10.1128/JVI.01459-15 26202227 PMC4577895

[B26] Hubel P. Urban C. Bergant V. Schneider W. M. Knauer B. Stukalov A. (2019). A protein-interaction network of interferon-stimulated genes extends the innate immune system landscape. *Nat. Immunol.* 20 493–502. 10.1038/s41590-019-0323-3 30833792

[B27] Ibsen M. S. Gad H. H. Andersen L. L. Hornung V. Julkunen I. Sarkar S. N. (2015). Structural and functional analysis reveals that human OASL binds dsRNA to enhance RIG-I signaling. *Nucleic Acids Res.* 43 5236–5248. 10.1093/nar/gkv389 25925578 PMC4446440

[B28] Itzhaki R. F. (2018). Corroboration of a major role for herpes simplex virus type 1 in Alzheimer’s Disease. *Front. Aging Neurosci.* 10:324. 10.3389/fnagi.2018.00324 PMC6202583 30405395

[B29] Jacobs S. R. Damania B. (2011). The viral interferon regulatory factors of KSHV: immunosuppressors or oncogenes? *Front. Immunol.* 2:19. 10.3389/fimmu.2011.00019 22566809 PMC3342017

[B30] Jacobs S. R. Gregory S. M. West J. A. Wollish A. C. Bennett C. L. Blackbourn D. J. (2013). The viral interferon regulatory factors of kaposi’s sarcoma-associated herpesvirus differ in their inhibition of interferon activation mediated by toll-like receptor 3. *J. Virol.* 87 798–806. 10.1128/JVI.01851-12 23115281 PMC3554052

[B31] Jacobs S. R. Stopford C. M. West J. A. Bennett C. L. Giffin L. Damania B. (2015). Kaposi’s sarcoma-associated herpesvirus viral interferon regulatory factor 1 interacts with a member of the interferon-stimulated gene 15 pathway. *J. Virol.* 89 11572–11583. 10.1128/JVI.01482-15 26355087 PMC4645652

[B32] Jenkins C. Abendroth A. Slobedman B. (2004). A novel viral transcript with homology to human interleukin-10 is expressed during latent human cytomegalovirus infection. *J. Virol.* 78 1440–1447. 10.1128/jvi.78.3.1440-1447.2004 14722299 PMC321375

[B33] Jeon Y. J. Yoo H. M. Chung C. H. (2010). ISG15 and immune diseases. *Biochim. Biophys. Acta* 1802 485–496. 10.1016/j.bbadis.2010.02.006 20153823 PMC7127291

[B34] Jiang Z. Su C. Zheng C. (2016). Herpes simplex virus 1 tegument protein UL41 counteracts IFIT3 antiviral innate immunity. *J. Virol.* 90 11056–11061. 10.1128/jvi.01672-16 27681138 PMC5126364

[B35] Jonsson K. L. Laustsen A. Krapp C. Skipper K. A. Thavachelvam K. Hotter D. (2017). IFI16 is required for DNA sensing in human macrophages by promoting production and function of cGAMP. *Nat. Commun.* 8:14391. 10.1038/ncomms14391 28186168 PMC5309897

[B36] Justesen J. Hartmann R. Kjeldgaard N. O. (2000). Gene structure and function of the 2′-5′-oligoadenylate synthetase family. *Cell Mol. Life Sci.* 57 1593–1612. 10.1007/pl00000644 11092454 PMC11146851

[B37] Kalamvoki M. Roizman B. (2014). HSV-1 degrades, stabilizes, requires, or is stung by STING depending on ICP0, the US3 protein kinase, and cell derivation. *Proc. Natl. Acad. Sci. U.S.A.* 111 E611–E617. 10.1073/pnas.1323414111 24449861 PMC3918790

[B38] Kim Y. J. Kim E. T. Kim Y. E. Lee M. K. Kwon K. M. Kim K. I. (2016). Consecutive inhibition of ISG15 expression and ISGylation by cytomegalovirus regulators. *PLoS Pathog* 12:e1005850. 10.1371/journal.ppat.1005850 27564865 PMC5001722

[B39] Koch S. Damas M. Freise A. Hage E. Dhingra A. Ruckert J. (2019). Kaposi’s sarcoma-associated herpesvirus vIRF2 protein utilizes an IFN-dependent pathway to regulate viral early gene expression. *PLoS Pathog* 15:e1007743. 10.1371/journal.ppat.1007743 31059555 PMC6522069

[B40] Lenschow D. J. (2010). Antiviral properties of ISG15. *Viruses* 2 2154–2168. 10.3390/v2102154 21994614 PMC3185569

[B41] Lenschow D. J. Lai C. Frias-Staheli N. Giannakopoulos N. V. Lutz A. Wolff T. (2007). IFN-stimulated gene 15 functions as a critical antiviral molecule against influenza, herpes, and Sindbis viruses. *Proc. Natl. Acad. Sci. U.S.A.* 104 1371–1376. 10.1073/pnas.0607038104 17227866 PMC1783119

[B42] Li T. Chen J. Cristea I. M. (2013). Human cytomegalovirus tegument protein pUL83 inhibits IFI16-mediated DNA sensing for immune evasion. *Cell Host Microbe* 14 591–599. 10.1016/j.chom.2013.10.007 24237704 PMC3876934

[B43] Lipps C. Badar M. Butueva M. Dubich T. Singh V. V. Rau S. (2017). Proliferation status defines functional properties of endothelial cells. *Cell Mol. Life Sci.* 74 1319–1333. 10.1007/s00018-016-2417-5 27853834 PMC11107763

[B44] Liu C. H. Zhou L. Chen G. Krug R. M. (2015). Battle between influenza A virus and a newly identified antiviral activity of the PARP-containing ZAPL protein. *Proc. Natl. Acad. Sci. US.A.* 112 14048–14053. 10.1073/pnas.1509745112 26504237 PMC4653199

[B45] Liu Q. Rao Y. Tian M. Zhang S. Feng P. (2019). Modulation of innate immune signaling pathways by herpesviruses. *Viruses* 11:E572. 10.3390/v11060572 31234396 PMC6630988

[B46] Liu X. Y. Chen W. Wei B. Shan Y. F. Wang C. (2011). IFN-induced TPR protein IFIT3 potentiates antiviral signaling by bridging MAVS and TBK1. *J. Immunol.* 187 2559–2568. 10.4049/jimmunol.1100963 21813773

[B47] Mao R. Nie H. Cai D. Zhang J. Liu H. Yan R. (2013). Inhibition of hepatitis B virus replication by the host zinc finger antiviral protein. *PLoS Pathog* 9:e1003494. 10.1371/journal.ppat.1003494 23853601 PMC3708887

[B48] Marques J. Anwar J. Eskildsen-Larsen S. Rebouillat D. Paludan S. R. Sen G. (2008). The p59 oligoadenylate synthetase-like protein possesses antiviral activity that requires the C-terminal ubiquitin-like domain. *J. Gen. Virol.* 89 2767–2772. 10.1099/vir.0.2008/003558-0 18931074

[B49] Mathers C. Schafer X. Martinez-Sobrido L. Munger J. (2014). The human cytomegalovirus UL26 protein antagonizes NF-kappaB activation. *J. Virol.* 88 14289–14300. 10.1128/JVI.02552-14 25275128 PMC4249132

[B50] Morales D. J. Lenschow D. J. (2013). The antiviral activities of ISG15. *J. Mol. Biol.* 425 4995–5008. 10.1016/j.jmb.2013.09.041 24095857 PMC4090058

[B51] Muller S. Moller P. Bick M. J. Wurr S. Becker S. Gunther S. (2007). Inhibition of filovirus replication by the zinc finger antiviral protein. *J. Virol.* 81 2391–2400. 10.1128/jvi.01601-06 17182693 PMC1865956

[B52] Myoung J. Ganem D. (2011). Generation of a doxycycline-inducible KSHV producer cell line of endothelial origin: maintenance of tight latency with efficient reactivation upon induction. *J. Virol. Methods* 174 12–21. 10.1016/j.jviromet.2011.03.012 21419799 PMC3095772

[B53] Orzalli M. H. Broekema N. M. Knipe D. M. (2016). Relative contributions of herpes simplex Virus 1 ICP0 and vhs to loss of cellular IFI16 Vary in Different Human Cell Types. *J. Virol.* 90 8351–8359. 10.1128/JVI.00939-16 27412599 PMC5008076

[B54] Orzalli M. H. Deluca N. A. Knipe D. M. (2012). Nuclear IFI16 induction of IRF-3 signaling during herpesviral infection and degradation of IFI16 by the viral ICP0 protein. *Proc. Natl. Acad. Sci. U.S.A.* 109 E3008–E3017. 10.1073/pnas.1211302109 23027953 PMC3497734

[B55] Page H. G. Read G. S. (2010). The virion host shutoff endonuclease (UL41) of herpes simplex virus interacts with the cellular cap-binding complex eIF4F. *J. Virol.* 84 6886–6890. 10.1128/JVI.00166-10 20427534 PMC2903273

[B56] Paijo J. Doring M. Spanier J. Grabski E. Nooruzzaman M. Schmidt T. (2016). cGAS senses human cytomegalovirus and induces type i interferon responses in human monocyte-derived cells. *PLoS Pathog* 12:e1005546. 10.1371/journal.ppat.1005546 27058035 PMC4825940

[B57] Pellett P. Roizman B. (2007). *Fields Virology*, 6th Edn Philadelphia: Lippincort, Williams, Wilkins, 2456.

[B58] Potter J. L. Narasimhan J. Mende-Mueller L. Haas A. L. (1999). Precursor processing of pro-ISG15/UCRP, an interferon-beta-induced ubiquitin-like protein. *J. Biol. Chem.* 274 25061–25068. 10.1074/jbc.274.35.25061 10455185

[B59] Rasmussen S. B. Sorensen L. N. Malmgaard L. Ank N. Baines J. D. Chen Z. J. (2007). Type I interferon production during herpes simplex virus infection is controlled by cell-type-specific viral recognition through Toll-like receptor 9, the mitochondrial antiviral signaling protein pathway, and novel recognition systems. *J. Virol.* 81 13315–13324. 10.1128/jvi.01167-07 17913820 PMC2168887

[B60] Schneider W. M. Chevillotte M. D. Rice C. M. (2014). Interferon-stimulated genes: a complex web of host defenses. *Annu. Rev. Immunol.* 32 513–545. 10.1146/annurev-immunol-032713-120231 24555472 PMC4313732

[B61] Schoggins J. W. (2019). Interferon-stimulated genes: what do they all do? *Annu. Rev. Virol*. 29 6 567–584. 10.1146/annurev-virology-092818-015756 31283436

[B62] Schoggins J. W. Macduff D. A. Imanaka N. Gainey M. D. Shrestha B. Eitson J. L. (2014). Pan-viral specificity of IFN-induced genes reveals new roles for cGAS in innate immunity. *Nature* 505 691–695. 10.1038/nature12862 24284630 PMC4077721

[B63] Schoggins J. W. Wilson S. J. Panis M. Murphy M. Y. Jones C. T. Bieniasz P. (2011). A diverse range of gene products are effectors of the type I interferon antiviral response. *Nature* 472 481–485. 10.1038/nature09907 21478870 PMC3409588

[B64] Shi H. X. Yang K. Liu X. Liu X. Y. Wei B. Shan Y. F. (2010). Positive regulation of interferon regulatory factor 3 activation by Herc5 via ISG15 modification. *Mol. Cell Biol.* 30 2424–2436. 10.1128/MCB.01466-09 20308324 PMC2863703

[B65] Smith C. Khanna R. (2013). Immune regulation of human herpesviruses and its implications for human transplantation. *Am. J. Transplant.* 13(Suppl. 3), 9–23. 10.1111/ajt.12005 23347211

[B66] Stempel M. Chan B. Brinkmann M. M. (2019). Coevolution pays off: Herpesviruses have the license to escape the DNA sensing pathway. *Med. Microbiol. Immunol*. 208 495–512. 10.1007/s00430-019-00582-0 30805724

[B67] Stoermer K. A. Morrison T. E. (2011). Complement and viral pathogenesis. *Virology* 411 362–373. 10.1016/j.virol.2010.12.045 21292294 PMC3073741

[B68] Strobl B. Bubic I. Bruns U. Steinborn R. Lajko R. Kolbe T. (2005). Novel functions of tyrosine kinase 2 in the antiviral defense against murine cytomegalovirus. *J. Immunol.* 175 4000–4008. 10.4049/jimmunol.175.6.4000 16148148

[B69] Su C. Zhang J. Zheng C. (2015). Herpes simplex virus 1 UL41 protein abrogates the antiviral activity of hZAP by degrading its mRNA. *Virol. J.* 12:203. 10.1186/s12985-015-0433-y 26625984 PMC4666169

[B70] Taddeo B. Roizman B. (2006). The virion host shutoff protein (UL41) of herpes simplex virus 1 is an endoribonuclease with a substrate specificity similar to that of RNase A. *J. Virol.* 80 9341–9345. 10.1128/jvi.01008-06 16940547 PMC1563938

[B71] Takata M. A. Gonçalves-Carneiro D. Zang T. M. Soll S. J. York A. Blanco-Melo D. (2017). CG dinucleotide suppression enables antiviral defence targeting non-self RNA. *Nature* 550:124. 10.1038/nature24039 28953888 PMC6592701

[B72] Tan J. C. Avdic S. Cao J. Z. Mocarski E. S. White K. L. Abendroth A. (2011). Inhibition of 2’,5’-oligoadenylate synthetase expression and function by the human cytomegalovirus ORF94 gene product. *J. Virol.* 85 5696–5700. 10.1128/JVI.02463-10 21450824 PMC3094971

[B73] Tang Q. Wang X. Gao G. (2017). The short form of the zinc finger antiviral protein inhibits influenza a virus protein expression and is antagonized by the virus-encoded NS1. *J. Virol* 91 e1909–e1916. 10.1128/JVI.01909-16 27807230 PMC5215320

[B74] Unterholzner L. Keating S. E. Baran M. Horan K. A. Jensen S. B. Sharma S. (2010). IFI16 is an innate immune sensor for intracellular DNA. *Nat. Immunol.* 11 997–1004. 10.1038/ni.1932 20890285 PMC3142795

[B75] Veeranki S. Choubey D. (2012). Interferon-inducible p200-family protein IFI16, an innate immune sensor for cytosolic and nuclear double-stranded DNA: regulation of subcellular localization. *Mol. Immunol.* 49 567–571. 10.1016/j.molimm.2011.11.004 22137500 PMC3249514

[B76] Vieira J. O’Hearn P. M. (2004). Use of the red fluorescent protein as a marker of Kaposi’s sarcoma-associated herpesvirus lytic gene expression. *Virology* 325 225–240. 10.1016/j.virol.2004.03.049 15246263

[B77] White K. L. Slobedman B. Mocarski E. S. (2000). Human cytomegalovirus latency-associated protein pORF94 is dispensable for productive and latent infection. *J. Virol.* 74 9333–9337. 10.1128/jvi.74.19.9333-9337.2000 10982383 PMC102135

[B78] Wu J. J. Li W. Shao Y. Avey D. Fu B. Gillen J. (2015). Inhibition of cGAS DNA sensing by a herpesvirus virion protein. *Cell Host Microbe* 18 333–344. 10.1016/j.chom.2015.07.015 26320998 PMC4567405

[B79] Xie L. Lu B. Zheng Z. Miao Y. Liu Y. Zhang Y. (2018). The 3C protease of enterovirus A71 counteracts the activity of host zinc-finger antiviral protein (ZAP). *J. Gen. Virol.* 99 73–85. 10.1099/jgv.0.000982 29182509

[B80] Zhang J. Das S. C. Kotalik C. Pattnaik A. K. Zhang L. (2004). The latent membrane protein 1 of Epstein-Barr virus establishes an antiviral state via induction of interferon-stimulated genes. *J. Biol. Chem.* 279 46335–46342. 10.1074/jbc.m403966200 15322136

[B81] Zhu J. Zhang Y. Ghosh A. Cuevas R. A. Forero A. Dhar J. (2014). Antiviral activity of human OASL protein is mediated by enhancing signaling of the RIG-I RNA sensor. *Immunity* 40 936–948. 10.1016/j.immuni.2014.05.007 24931123 PMC4101812

[B82] Zhu M. John S. Berg M. Leonard W. J. (1999). Functional association of Nmi with Stat5 and Stat1 in IL-2- and IFNgamma-mediated signaling. *Cell* 96 121–130. 10.1016/s0092-8674(00)80965-4 9989503

[B83] Zhu Y. Chen G. Lv F. Wang X. Ji X. Xu Y. (2011). Zinc-finger antiviral protein inhibits HIV-1 infection by selectively targeting multiply spliced viral mRNAs for degradation. *Proc. Natl. Acad. Sci. U.S.A.* 108 15834–15839. 10.1073/pnas.1101676108 21876179 PMC3179061

[B84] Zimmermann A. Trilling M. Wagner M. Wilborn M. Bubic I. Jonjic S. (2005). A cytomegaloviral protein reveals a dual role for STAT2 in IFN-{*gamma*} signaling and antiviral responses. *J. Exp. Med.* 201 1543–1553. 10.1084/jem.20041401 15883169 PMC2212917

